# Decolonization of gastrointestinal carriage of vancomycin-resistant *Enterococcus faecium*: case series and review of literature

**DOI:** 10.1186/1471-2334-14-514

**Published:** 2014-09-23

**Authors:** Vincent CC Cheng, Jonathan HK Chen, Josepha WM Tai, Sally CY Wong, Rosana WS Poon, Ivan FN Hung, Kelvin KW To, Jasper FW Chan, Pak-Leung Ho, Chung-Mau Lo, Kwok-Yung Yuen

**Affiliations:** Department of Microbiology, Queen Mary Hospital, Hong Kong Special Administrative Region, Hong Kong, China; Infection Control Team, Queen Mary Hospital, Hong Kong Special Administrative Region, Hong Kong, China; Department of Medicine, Queen Mary Hospital, Hong Kong Special Administrative Region, Hong Kong, China; Department of Surgery, Queen Mary Hospital, Hong Kong Special Administrative Region, Hong Kong, China

**Keywords:** Vancomycin-resistant enterococci, Decolonization, Bowel preparation, Polyethylene glycol, Linezolid, Daptomycin, *Lactobacillus rhamnosus* GG

## Abstract

**Background:**

Prolonged asymptomatic carriage of vancomycin-resistant enterococci (VRE) in the gastrointestinal tract and the lack of effective decolonization regimen perpetuate the endemicity of VRE in the healthcare settings.

**Case presentation:**

We report a regimen for decolonization of gastrointestinal carriage of VRE by a combination of environmental disinfection, patient isolation, bowel preparation to wash-out the fecal bacterial population using polyethylene glycol, a five-day course of oral absorbable linezolid and non-absorbable daptomycin to suppress any remaining VRE, and subsequent oral *Lactobacillus rhamnosus* GG to maintain the colonization resistance in four patients, including two patients with end-stage liver cirrhosis, one patient with complication post liver transplant, and one patient with complicated infective endocarditis. All patients had clearance of VRE immediately after decolonization, and 3 of them remained VRE-free for 23 to 137 days of hospitalization, despite subsequent use of intravenous broad-spectrum antibiotics without anti-VRE activity.

**Conclusion:**

This strategy should be further studied in settings of low VRE endemicity with limited isolation facilities.

**Electronic supplementary material:**

The online version of this article (doi:10.1186/1471-2334-14-514) contains supplementary material, which is available to authorized users.

## Background

Vancomycin-resistant enterococci (VRE) has been endemic in many parts of the word. Prolonged asymptomatic carriage of VRE in the gastrointestinal tract and the lack of effective decolonization regimen perpetuate the endemicity of VRE in the healthcare settings [1]. In Hong Kong, an increasing number of sporadic cases of VRE are observed, with a prevalence of 0.32% of admission episodes by active surveillance culture [2]. We adopted a proactive infection measures by the implementation of active surveillance culture, extensive contact tracing, isolation of VRE-positive patients and environmental disinfection to control nosocomial transmission of VRE [2–4]. However, the limited availability of single rooms fails to match with the increasing number of patients colonized with VRE in recent years. Therefore, an effective regimen for VRE decolonization is necessary in resource-limited areas like Hong Kong. Here, we report a protocol of VRE decolonization in four patients, including two patients with end-stage liver cirrhosis awaiting liver transplantation, one patient with a complicated post-liver transplant recovery, and one patient with complicated infective endocarditis. Decolonization of VRE is crucial in liver transplant candidates as VRE colonization is associated with an increased risk of infection and death among liver transplant recipients [5]. In contrast to the previous attempt of VRE decolonization using only antimicrobial agents or probiotics, we include the use of polyethylene glycol for bowel preparation to wash-out the fecal bacterial population prior to the administration of oral linezolid and daptomycin. *Lactobacillus rhamnosus* GG is then given to maintain the colonization resistance after antimicrobial decolonization. Our aim of VRE decolonization was to reduce the risk of subsequent invasive VRE infection among the high-risk patients, and to limit the risk of nosocomial transmission of VRE in our locality.

## Case presentation

### Decolonization of gastrointestinal carriage of VRE

**Figure 1:**
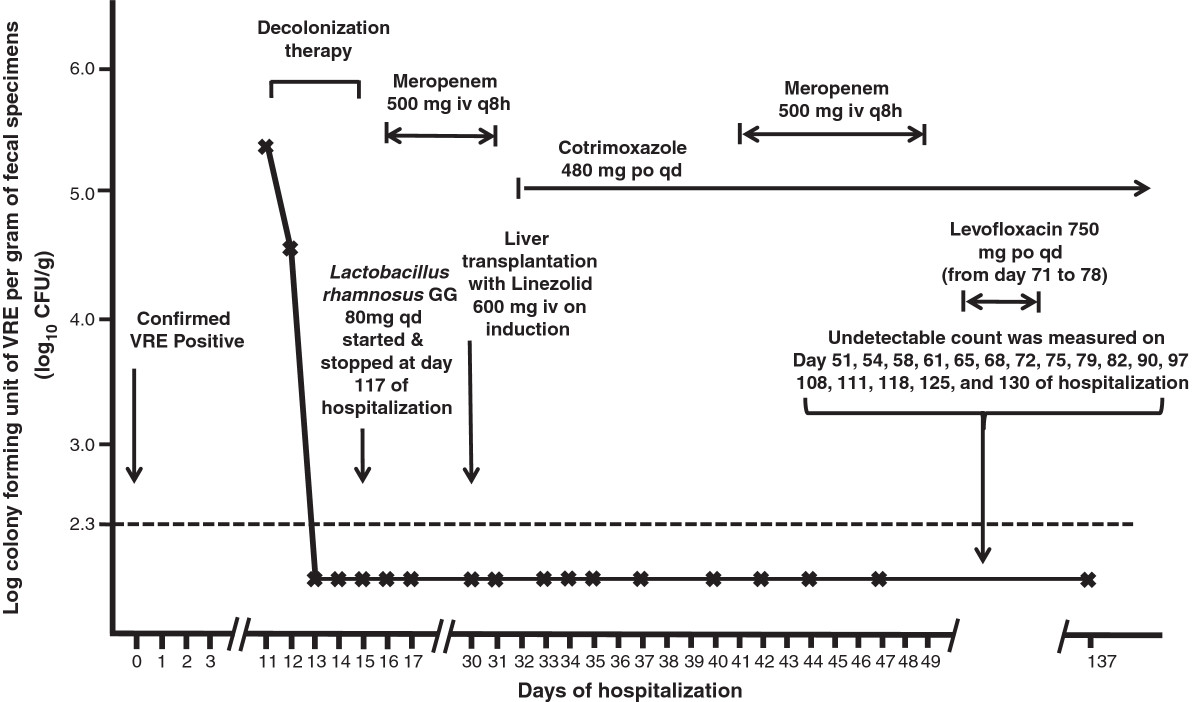
**Serial quantitative culture of gastrointestinal carriage of vancomycin**-**resistant** ***Enterococcus faecium*** **and concomitant use of antimicrobial agents in case 1.** Note. Intravenous meropenem 500 mg every 8 hourly was given between day 16 and 31 for recurrent isolation of extended-spectrum β-lactamase-producing *Klebsiella species* in sputum; intravenous meropenem 500 mg every 8 hourly was given again between day 41 and 49 for low grade fever without microbiological documentation of infection; oral levofloxacin 750 mg daily was given between day 71 and 78 for urine isolation of extended-spectrum β-lactamase-producing *Klebsiella species*; oral cotrimoxazole 480 mg twice daily was given after liver transplantation as pre-emptive prophylactic agent. The dotted horizontal line denoted the detection limit of VRE in fecal samples by broth enrichment ~ 200 cfu/g (2.3 log_10_ cfu/g).

**Figure 2:**
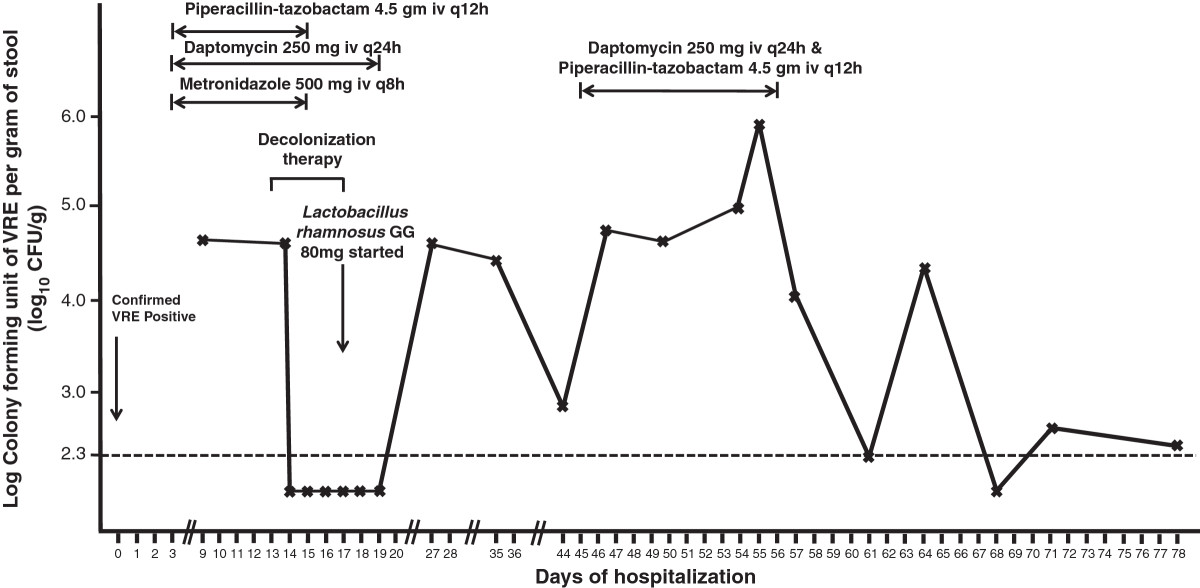
**Serial quantitative culture of gastrointestinal carriage of vancomycin**-**resistant** ***Enterococcus faecium*** **and concomitant use of antimicrobial agents in case 2.** Note. Intravenous piperacillin-tazobactam 4.5 gm iv q12h, daptomycin 250 mg iv q12h, and metronidazole 500 mg iv q8h were given from day 3 for empirical treatment of spontaneous bacterial peritonitis, while intravenous piperacillin-tazobactam 4.5 gm iv q12h, and daptomycin 250 mg iv q12h were given between day 45 and 56 for clinical diagnosis of another episode of spontaneous bacterial peritonitis. The dotted horizontal line denoted the detection limit of VRE in fecal samples by broth enrichment ~ 200 cfu/g (2.3 log10 cfu/g).

**Figure 3:**
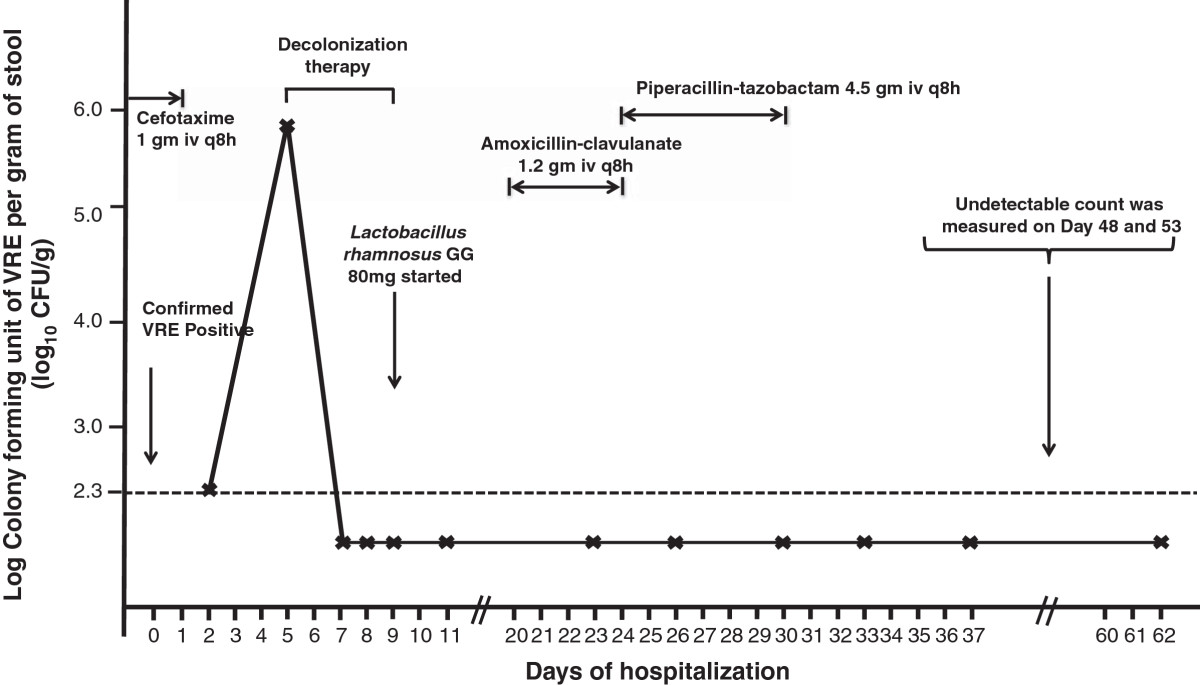
**Serial quantitative culture of gastrointestinal carriage of vancomycin**-**resistant** ***Enterococcus faecium*** **and concomitant use of antimicrobial agents in case 3.** Note. Intravenous cefotaxime 1 gm iv q8h was given from the referral hospital and stopped on day 1 of hospitalization. Amoxicillin-clavulanate was given on day 20 and stepped up to piperacillin-tazobactam on day 24 of hospitalization for nosocomial onset of fever of unknown source. The dotted horizontal line denoted the detection limit of VRE in fecal samples by broth enrichment ~ 200 cfu/g (2.3 log_10_ cfu/g).

**Figure 4:**
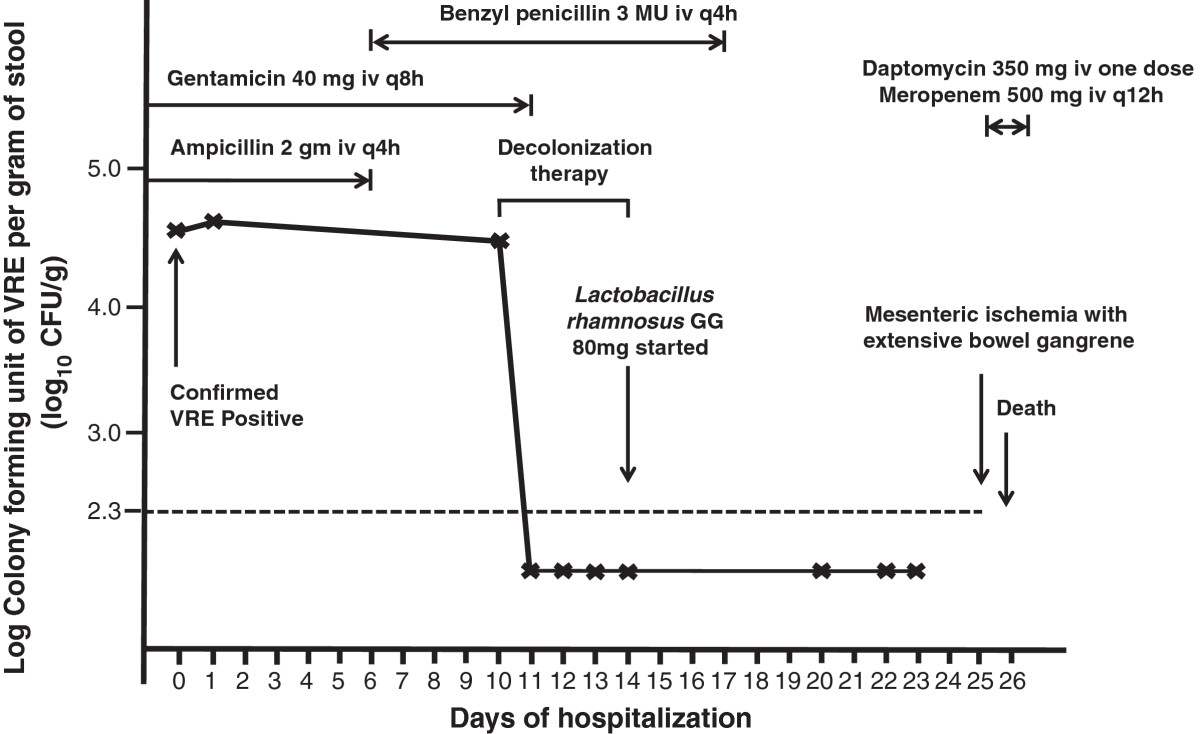
**Serial quantitative culture of gastrointestinal carriage of vancomycin**-**resistant** ***Enterococcus faecium*** **and concomitant use of antimicrobial agents in case 4.** Note. Intravenous ampicillin 2 gm iv q8h, and gentamicin 40 mg iv q8h were given from the referral hospital for treatment of infective endocarditis due to viridans streptococci. Antibiotic was changed to benzyl penicillin 3 MU iv q4h on day 7 upon clinical consultation to microbiologist, and stopped on day 17 of hospitalization after completion of treatment. Meropenem 500 mg iv q12h and one dose of daptomycin 350 mg iv was given on day 25 for systemic sepsis complicating mesenteric ischemia and bowel gangrene. Patient succumbed on day 26 of hospitalization despite maximal supportive therapy. The dotted horizontal line denoted the detection limit of VRE in fecal samples by broth enrichment ~ 200 cfu/g (2.3 log_10_ cfu/g).

Case 1: A 59-year-old man was referred to the Liver Transplant Unit of Queen Mary Hospital, a university-affiliated hospital with 1,600 bed, for consideration of cadaveric liver transplantation due to chronic hepatitis B-related end-stage cirrhosis on 14 September 2013. Queen Mary Hospital is the only liver transplant center in Hong Kong, with approximately 80 patients undergoing liver transplantation per year. Upon admission, rectal swab with visible stool content was collected for the detection of multidrug-resistant organisms including VRE in accordance to the hospital infection control policy. Briefly, patients with history of hospitalization or receiving surgical operation outside Hong Kong within the past 12 months before admission or patients with history of admissions to other local hospitals within the past 3 months were included in the active surveillance [2, 4, 6]. The patient was confirmed to be positive for vancomycin-resistant *Enterococcus faecium* which was resistant to ampicillin, chloramphenicol, nitrofurantoin, levofloxacin, minocycline, rifampicin, tetracycline, fosfomycin, high-level gentamicin, and high-level streptomycin. The minimal inhibitory concentration (MIC) of vancomycin was >256 μg/ml. *VanA* gene was detected by polymerase chain reaction (PCR). The MIC of linezolid and daptomycin was 1.5 μg/ml and 3.0 μg/ml respectively, while the MIC breakpoint of linezolid and daptomycin was ≤ 2 μg/ml and ≤ 4 μg/ml for enterococcus species respectively with reference to Clinical and Laboratory Standards Institute. Multilocus sequence typing (MLST) demonstrated that the strain was ST761 (profile 70-1-1-1-12-1-1), which is a member of the pandemic clonal complex 17 lineage. The patient was isolated in a single room with strict contact precautions since day 2 of hospitalization. Healthcare workers wore gloves and gowns during patient care practice. Alcohol-based hand rub was provided in the isolation room for hand hygiene. Dedicated medical items such as stethoscope, thermometer, and blood pressure cuff were available and solely used on this patient. Decolonization of VRE was performed between day 11 and 15 of hospitalization according to our protocol (Table 1). The bacterial load of VRE in rectal swabs with visible stool content was monitored daily. The VRE counts decreased from >2×10^5^ colony forming units per gram (cfu/g) of stool (baseline on day 11) to 3.4×10^4^ cfu/g of stool on day 12 and undetectable (<200 cfu/g) on day 13. Despite the administration of broad-spectrum antibiotics after successful decolonization, the serial rectal swabs remained negative for VRE in vancomycin and clindamycin containing enterococcal enrichment broth culture (Figure 1). ABO blood group matched-cadaveric liver graft became available on day 30 of hospitalization and transplantation was performed under coverage with intravenous linezolid 600 mg 30 minutes before surgical incision, and one dose postoperatively. The operation lasted for 11 hours without immediate complications. Patient was managed in the adult intensive care unit during the postoperative period and transferred to the liver transplant ward on day 32 of hospitalization (post-operative day 2). Serial rectal swabs were collected on day 1, 3, 4, 5, 7, 10, 12, 14, and 17 after liver transplantation, thereafter twice weekly until day 66, and weekly until day 107 after liver transplantation (day 137 of hospitalization) (Figure 1). No growth of VRE was detected in broth enrichment culture.Case 2: A 56-year-old man had living-donor liver transplantation for early stage of moderately differentiated hepatocellular carcinoma on 31 July 2012. He developed recurrent ascites as a result of portal vein and hepatic vein stenosis 6 months post-transplant requiring multiple episodes of hospitalization. On 26 February 2014, he was detected to have gastrointestinal colonization of VRE and was subjected to VRE decolonization after 13 days of hospitalization (Figure 2). However, patient had poor tolerance to oral ingestion of polyethylene glycol for bowel preparation due to refractory ascites. Since the procedure of decolonization was started, it was continued despite insufficient bowel preparation. Patient had transient suppression of VRE between day 14 and 19 and VRE relapsed on day 27 of hospitalization. The decolonization regimen was not successful in this case with inadequate bowel preparation.Case 3: A 44-year-old man was transferred from a regional hospital to the Liver Transplant Unit of Queen Mary Hospital for consideration of cadaveric liver transplantation due to hepatitis B-related hepatic failure on 15 March 2014. He was found to have gastrointestinal carriage of VRE upon admission screening. While waiting for a potential liver graft, VRE decolonization was initiated on day 5 of hospitalization. He remained VRE negative on serial monitoring till day 62 of hospitalization despite intermittent use of antibiotics (Figure 3).Case 4: A 71-year-old-lady was transferred from a regional hospital to the Cardiothoracic Surgical Unit, Queen Mary Hospital, for consideration of surgical intervention for viridans streptococci-related infective endocarditis complicated with cardiac failure. She had history of chronic rheumatic heart disease with mitral stenosis, mitral regurgitation, aortic regurgitation, and tricuspid regurgitation. She was noted to have gastrointestinal carriage of VRE upon admission screening, and was put on VRE decolonization as per protocol on day 10 of hospitalization. Serial rectal swabs culture remained VRE negative (broth enrichment culture) till day 23 of hospitalization (Figure 4). However, patient developed respiratory distress, metabolic acidosis, hypotension, oliguric renal impairment, and radiological appearance of dilated bowel loops suggestive of mesenteric ischemia on day 25. Urgent exploratory laparotomy revealed the presence of 300 ml foul-smelling, blood-stained peritoneal fluid with gangrenous change of bowel from duodenojejunal flexure to mid-sigmoid colon. Despite empirical use of meropenem and daptomycin, inotropic and ventilator support, patient succumbed on day 26 of hospitalization. No VRE could be detected in all her clinical specimens or serial rectal swabs after decolonization.

**Table 1:** Protocol of vancomycin-resistant enterococci decolonization*

1.	Patient was managed in isolation room A and subjected to bowel preparation according to the protocol commonly used prior to colonoscopy examination: (i) ingestion of 2 liters of polyethylene glycol (Klean prep) over 6 hours to wash out the bowel content; (ii) taking fluid diet including rice water, clear soup, and fruit juice on the first day of decolonization.
2.	When the defecated bowel content became clear fluid, patient was transferred from isolation room A to B, which had been terminally disinfected with sodium hypochlorite 1,000 ppm.
3.	After transferal to isolation room B, a five-day course of medication with activity against VRE was given, including oral linezolid 600 mg every 12 hourly, orally-taken intravenous preparation of daptomycin 8 mg per kg daily.
4.	At the same time, the patient was cleansed with 4% chlorhexidine bath and shampoo, and oral chlorhexidine gargle for 5 days. Where possible, avoid use of other antibiotics treatment during the decolonization period.
5.	At the time of bathing, the patient’s clothes, underwear, and bed linens were replaced and sent for hot laundry daily. All personal belongings were disinfected to prevent re-colonization. The isolation room was thoroughly cleaned and disinfected by sodium hypochlorite 1,000 ppm twice daily.
6.	After completion of 5-day decolonization regimen, *Lactobacillus rhamnosus* GG 80 mg was given daily to replace the gut flora.
7.	All foods and drinks throughout the decolonization procedure must be boiled. All visitors and healthcare workers must comply with hand hygiene with alcohol based hand rub.

Note. VRE, vancomycin-resistant enterococci; *The decolonization protocol was designed and coordinated by the infection control team for all patients in Queen Mary Hospital. Infection control nurses closely monitored and audited the compliance of the procedures required for decolonization.

### Microbiological analysis

For VRE screening, stool or rectal swabs with visible fecal components were inoculated onto chromogenic agar (chromID VRE, bioMerieux, France) and incubated aerobically at 35°C for 48 hours for identification by Vitek 2 automated identification system (bioMerieux, France) as previously described [4]. The Kirby-Bauer disk diffusion method and E-test (AB Biodisk, Solna, Sweden) were used to determine the antimicrobial susceptibility of the enterococci. Isolates with potential vancomycin resistance were proceeded to *vanA* gene PCR and MLST as previously described [4]. For VRE bacterial load quantitation, around 1 g of fecal sample was suspended into 2 ml normal saline and serially diluted suspensions were plated onto chromogenic agar. In addition, broth enrichment was performed by inoculating another 10 μl aliquot of the fecal suspension into the vancomycin containing brain-heart-infusion broth. The broth was incubated at 37°C overnight and further subcultured onto chromogenic agar in 35°C aerobic incubation for 48 hours. The lower detection limit of this method is 200 cfu/g.

## Discussion

Decolonization of VRE poses a great challenge for clinical microbiologists and infection control professionals. It is difficult to suppress the fecal burden of enterococci, ranging from 1–10 million cfu/g of stool, within the gastrointestinal tract by sole use of antimicrobial agents that are only bacteriostatic. We aim to overcome the problem of significant enterococci burden by mechanical removal of fecal load before the administration of high concentration of topical antimicrobial agents. Therefore, in addition to systemic and topical antibiotics, we adopt a protocol for bowel wash-out commonly used prior to colonoscopy to remove the majority of the fecal bacterial burden, and combined it with adjunctive infection control measures and probiotic. To our knowledge, the use of polyethylene glycol for bowel preparation in the aim to eliminate fecal bacterial population has not been attempted in previous eradication protocols for VRE or other multiple drug resistant bacteria. We have successfully eradicated VRE carriage in a high-risk patient with end-stage cirrhosis before cadaveric liver transplantation. This patient was chosen as the first case in our pilot study because decolonization of VRE is crucial in liver transplant candidates. In one study, it was found that liver transplant candidates and recipients with VRE colonization had an increased risk of VRE infection (adjusted odd ratio 3.61, 95% CI 2.01-6.47) and of death (adjusted odd ratio 2.12, 95% CI 1.27-3.54) compared with non-colonized patients [5].

In the literature, antimicrobial agents with high luminal concentration had been used to decolonize gastrointestinal carriage of VRE in 142 patients (Table 2). Bacitracin-containing regimens given orally or via gastrostomy tube for 10 days to 29 days were most common, and was used in 76 (54%) of 142 patients [7–12]. The results were rather variable, while the overall observed rate of VRE clearance was found to be 43% to 100% in patients treated with bacitracin-containing regimens [7, 9, 11], long term follow up showed that VRE free was only observed in 33% to 53% at 3 weeks post-treatment [8, 10]. In one study using a combination of oral bacitracin and doxycycline as VRE eradication regimen, although quantitation of VRE in stool showed an initial 3-log reduction of VRE count per gram of stool during the 2-week therapy, the VRE count in stool increased to pretreatment levels of 7.8 and 7.4 log_10_/g at 2–4 and 5–7 weeks post-treatment respectively [9]. In another randomized placebo controlled study to assess the use of 10-day course of zinc bacitracin capsules in enteric eradication of VRE, only 2 (33%) of 6 patients in each group were VRE negative after 3 weeks suggesting that there was no difference in eradication rate of VRE in the zinc bacitracin and the control group [10]. In a later study, combination of oral bacitracin and gentamicin was evaluated. After 3 months of follow up, only 5 (18%) of 28 patients had no VRE isolated from stool cultures. Antimicrobial side effects were a major concern. Of the 45 patients originally enrolled on the treatment arm, 17 (38%) could not tolerate bacitracin and gentamicin because of nausea, vomiting, and diarrhea [12]. Ramoplanin, an orally administered lipoglycodepsipeptide antibiotic that is not absorbed systemically, has in vitro activity against vancomycin-resistant *E. faecium* and *E. faecalis*, and has been shown to suppress gastrointestinal colonization of VRE in up to 90% of patients during the course of ramoplanin in a phase II, double-blinded, randomized, multicenter, placebo-controlled study. However, recurrence of VRE colonization was observed in most patients 14 days after treatment [13, 14]. Short courses of novobiocin in combination with tetracycline or doxycycline were also ineffective in eradicating gastrointestinal carriage of VRE [15].

**Table 2:** **Summary of gastrointestinal decolonization of vancomycin**-**resistant Enterococcus by antimicrobial therapy**

Ref	Country/year of publication/study setting	Decolonization regimens/study end point(if mentioned)	Results at study end point	Microbiology culture methods/presence of broth enrichment or not
[7]	US/1994/observational study	Oral vancomycin 125 mg q6h for 10 days^a^/follow up for 15 days post treatment	VRE negative in 8 (42%) of 19 patients	Campylobacter agar containing 10 μg/ml of vancomycin (B-D Microbiology Systems, Cockeysville, MD, USA)/no broth enrichment
[7]	US/1994/observational study	Oral bacitracin 25,000 U (500 mg) q6h for 10 days/follow up for 15 days post treatment	VRE negative in 8 (100%) of 8 patients	Campylobacter agar containing 10 μg/ml of vancomycin (B-D Microbiology Systems, Cockeysville, MD, USA)/no broth enrichment
[15]	US/1995/observational study	Oral novobiocin (500 mg q6h plus oral tetracycline 500 mg q6h (five patients) or intravenous doxycycline 100 mg q12h (one patient) for a median of 3.5 days (range, 1 to 6 days)	VRE negative in only one patient while receiving decolonization therapy	Not mentioned
[8]	US/1995/observational study	Bacitracin 25,000 U (diluted in 5 mL of 0.9% normal saline) given orally or by gastrostomy tube twice a day for 10 days/follow up for 3 weeks post treatment	VRE negative in 5 (63%) of 8 patients	Not mentioned
[9]	Canada/1999/prospective observational cohort study in a tertiary care institution	Oral doses of bacitracin solution (75,000 U/15 mL) four times daily and doxycycline 100 mg once daily for 14 days/follow up for 4 months	VRE negative in 15 (100%) of the antibiotic treated vs 8 (33.3%) of the untreated patients (P <0 .001) at the end of treatment; but VRE positive in 9 (60%) of 15 and 15 (62.5%) of 24 in the treated and untreated cohort (p = 0.86) following up for a mean of 127 and 130 days respectively ^b^	M-enterococcal agar with vancomycin (6 mg/mL)/no broth enrichment
[10]	US/2001/randomized, controlled study	Oral zinc bacitracin (50,000 U) q6h for 10 days vs placebo/follow up for 3 weeks post treatment	VRE negative in 2 (33%) of 6 patients in each group after 3 weeks post treatment	Bile esculin agar plates supplemented with 6 mg/mL of vancomycin/no broth enrichment
[14]	US/2001/phase II, double-blinded, randomized, multicenter, placebo-controlled study	Oral ramoplanin: 2 daily doses of 100 mg or 400 mg or placebo for 7 days/follow up on day 0, 7, and 14 post treatment	Day 0: VRE negative in 17 (81%) of 21 and 18 (90%) of 20 patients in the 100-mg and 400-mg ramoplanin groups; Day 7: VRE negative in 6 (29%) of 21 and 7 (41%) of 17 in the 100-mg and 400-mg ramoplanin groups; Day 14: VRE negative in 4 (21%) of 19 and 5 (29%) of 17 patients in the 100-mg and 400-mg ramoplanin groups^c^	Bile-esculin azide broth that contained 6 mg/mL of vancomycin (Hardy Diagnostics)/broth enrichment
[12]	US/2002/observational study	Oral bacitracin (25,000 U three times daily) and oral gentamicin (80 mg three times daily) for a mean duration of 16 days (median, 14 days; range, 7 to 29 days)/follow up for 3 months post treatment	VRE negative in 5 (17.8%) of 28 patients	Not mentioned
[11]	France/2010/observational study in a geriatric rehabilitation care facility	Oral bacitracin 30,000 U three times daily for 15 days/follow up for 6 months	VRE negative in 3 (43%) of 7 patients at the end of therapy and at 6 months	Not mentioned
[11]	France/2010/observational study in a geriatric rehabilitation care facility	Bacitracin 30,000 U three times daily plus streptomycin 1 g once daily orally for 15 days/follow up for 6 months	VRE negative in 3 (75%) of 4 patients at the end of therapy and at 6 months	Not mentioned

Note. U, units; VRE, vancomycin-resistant enterococci; ^a^high intraluminal concentrations attained with oral vancomycin administration greatly exceed the MICs of VRE; ^b^Quantitative VRE stool cultures in the treated cohort revealed an initial 3.1 log_10_/g decrease, but there was an increase to pretreatment levels of 7.8 and 7.4 log_10_/g at 2–4 and 5–7 weeks post-treatment respectively; ^c^For placebo group, VRE negative in none of 20 patients in day 0, 2 (10%) of 20 patients in day 7, and 5 (25%) of 20 patients in day 14 post treatment.

With reference to the suboptimal VRE eradication outcomes in the literature when antimicrobial treatment was used alone without fecal wash-out, we attempted to reduce the microbial burden of the gastrointestinal tract by performing bowel wash-out prior to antimicrobial treatment. In addition, we used a new combination of a 5-day course of oral linezolid with systemic effect and oral non-absorbable daptomycin with topical effect to maximize the anti-VRE activity in the gastrointestinal tract. Daptomycin oral therapy had previously been given with minimal systemic absorption [16]. Oral absorbable linezolid may help to decrease VRE counts in biliary or urinary system, but even systemic antibiotics may not work if patients have stone or parasitic nidus in the biliary or urinary tract associated with biofilm formation which can be colonized by VRE. Thus organ imaging to exclude these kinds of foci would be important before instituting the decolonization. This regimen successfully reduced the quantitative bacterial count of VRE from 10^4^ - 10^5^ cfu/g of stool to undetectable level within 3 days of decolonization therapy in at least 3 patients (case 1, case 3, and case 4). However, our regimen failed in one patient (case 2) who could not tolerate oral ingestion of polyethylene glycol for bowel preparation due to refractory ascites. We believe that complete fecal wash-out is the key component of our VRE decolonization regimen.

Adjunctive infection control measures are essential to prevent re-acquisition of VRE from patients’ skin and hospital environment during the process of decolonization. Preparation of 4% chlorhexidine gluconate bath and shampoo was used to reduce the cutaneous colonization of VRE as previously described [17]. Thorough environmental disinfection by sodium hypochlorite 1,000 ppm was done twice daily. At the time of bathing, the patient’s clothes, underwear, and bed linens were replaced and sent for hot laundry daily. Probiotic *Lactobacillus rhamnosus* GG 80 mg daily for repopulating the gram positive anaerobic flora was used after the 5-day course of linezolid and daptomycin to consolidate the effect of decolonization and may improve the colonization resistance against re-colonization by VRE. In fact, use of *Lactobacillus rhamnosus* GG or *Lactobacillus rhamnosus* Lcr35 had been attempted to decolonize 49 patients in the literature with variable results (Table 3). A randomized placebo-controlled study using standard dose of *Lactobacillus* for 3 weeks in children showed that rectal swab was rendered negative for VRE at the end of therapy in 63% of patients using semi-quantitative method without broth enrichment, but the difference in VRE carrier state was no longer significant 1 week after completion of treatment [18], suggesting that the effect of *Lactobacillus* alone may be temporary. Furthermore, using probiotic containing *Lactobacillus species* alone may not be feasible in patient receiving antibiotics [19]. In contrast, with the appropriate use of fecal wash-out, antimicrobial agents, and *Lactobacillus rhamnosus GG*, our patients appeared to maintain a persistent VRE negative status, tested by broth enrichment culture of fecal samples, despite the use of broad-spectrum antibiotics after the decolonization.

**Table 3:** **Summary of gastrointestinal decolonization of vancomycin**-**resistant enterococci by probiotic therapy**

Ref	Country/year of publication/study setting	Decolonization regimens/study end point(if mentioned)	Results at study end point	Microbiology culture methods/presence of broth enrichment or not
[20]	Australia/2007/double-blind, randomized, placebo-controlled trial in nephrology patients	*Lactobacillus rhamnosus* GG in the form of commercially available yoghurt: 100 g daily of yoghurt containing *Lactobacillus rhamnosus* GG for 4 weeks/follow up for 4 weeks post treatment	VRE negative in all 11 patients in treatment group at the end of therapy; 8 (73%) remained VRE negative 4 weeks post treatment; VRE negative in 1 (8%) of 12 control patient at the end of treatment	Enterococcosel agar (BD, Sparks, Md, USA) containing 6 μg vancomycin/no broth enrichment
[21]	France/2010/double-blind randomized pilot study in adult	*Lactobacillus rhamnosus* Lcr35: 5-week course of Lcr35 (10^9^ active cells daily) or a placebo/follow up till the end of therapy	VRE negative in 3 (50%) of 6 patients in treatment group vs 2 (100%) of 2 patients in control group at the end of therapy	Not mentioned
[18]	Poland/2011/randomized, single-blind, placebo-controlled study in children	*Lactobacillus rhamnosus* GG 3 billion colony forming unit per day vs placebo for 21 days/follow up till the end of therapy	VRE negative in 20 (63%) of 32 patients in treatment group vs 7 (24%) of 29 in control group (p = 0.002)	Selective medium (D-Coccosel agar, BioMe’rieux) and a chromogenic medium (ChromID, BioMe’rieux)/no broth enrichment

Note: VRE, vancomycin-resistant enterococci.

The major limitation in this study is that the experience with our decolonization regimen was restricted to four patients to date. Additionally, there are potential risks and discomfort associated with the use of polyethylene glycol for fecal wash-out. Patients with severe renal impairment may have a risk of fluid retention and electrolyte disturbance. Patients with refractory ascites may not be able to tolerate the volume of polyethylene glycol. Inadequate bowel preparation may subsequently result in failure of VRE decolonization, like case 2 in our series. Further clinical trials should be performed to validate the effectiveness of this regimen and assess the potential development of antimicrobial resistance, as linezolid and daptomycin will be increasingly used in hospitals with rising incidence of infections due to resistant organisms [22, 23]. Stool concentration of oral linezolid and daptomycin can be done to compare against the measured MICs of the VRE isolates. However, we believe that the findings in this study are significant, where high-risk patient (case 1) with critical underlying co-morbidities has remained VRE negative for over 100 days after decolonization, even with subsequent use of broad-spectrum antibiotic therapy. Cost-effective analysis should be conducted in subsequent studies to measure the expenditure in VRE decolonization and medical management of invasive VRE infections. In addition, VRE decolonization on an outpatient basis, such as patient’s own residence, may be explored, especially in resource-poor regions where prolonged hospitalization for the purpose of decolonization may not be feasible.

## Conclusion

Our experience revealed that sustained decolonization of gastrointestinal carriage of VRE was possible by a combination of environmental disinfection, patient isolation, bowel preparation to wash-out the fecal bacterial population using polyethylene glycol, a five-day course of oral absorbable linezolid and non-absorbable daptomycin to suppress any remaining VRE, and subsequent oral *Lactobacillus rhamnosus* GG to maintain the colonization resistance. This strategy should be further studied in settings of low VRE endemicity with limited isolation facilities.

### Consent

Written informed consent was obtained from the four patients for publication of this case report and any accompanying images. A copy of the written consent is available for review by the Editor of this journal.

### Ethics statement

This study has been approved by the Institutional Review Board of the University of Hong Kong/Hospital Authority Hong Kong West Cluster.

## Authors’ original submitted files for images

Below are the links to the authors’ original submitted files for images. Authors’ original file for figure 1 Authors’ original file for figure 2 Authors’ original file for figure 3 Authors’ original file for figure 4
