# Supplementary figures and images for: Decolonization of gastrointestinal carriage of vancomycin-resistant Enterococcus faecium: case series and review of literature
Source: BMC Infect Dis. 2014 Sep 23;14:514. doi: 10.1186/1471-2334-14-514 (PMC4180964; doi:10.1186/1471-2334-14-514)

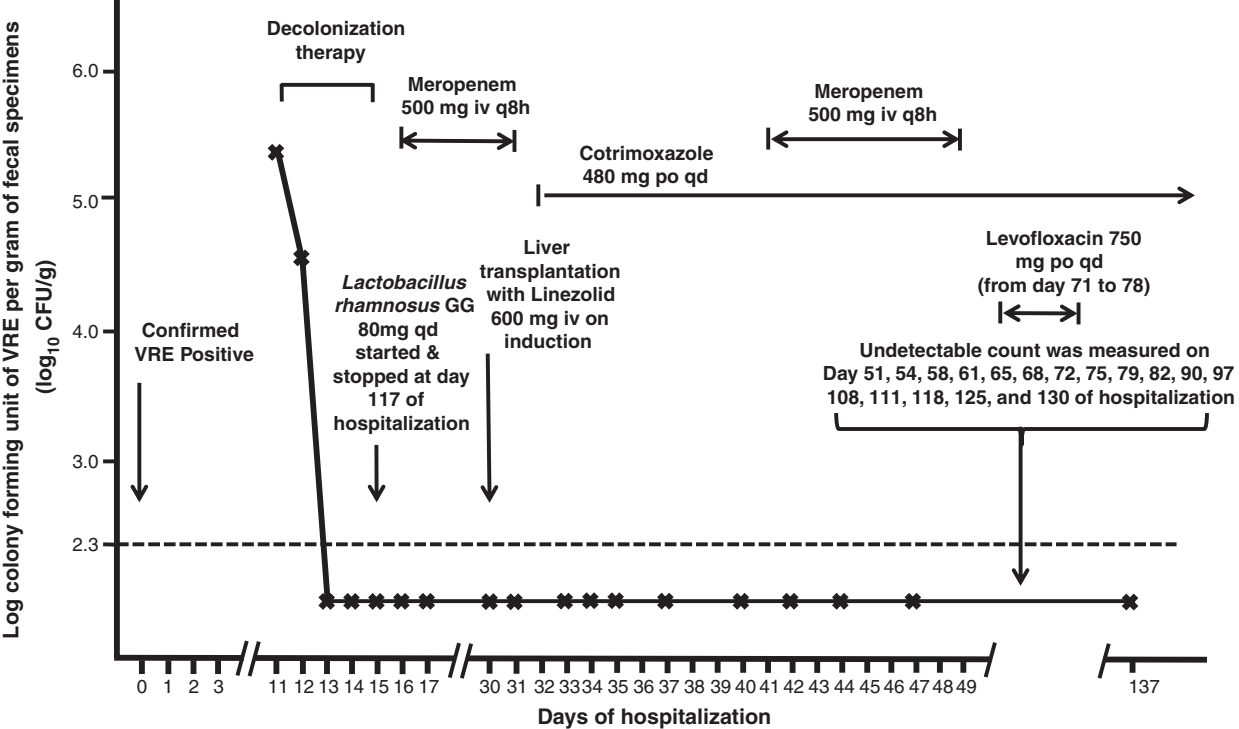

Supplement: Supplementary file 1 — Authors’ original file for figure 1 [file 12879_2014_3833_MOESM1_ESM.pdf]

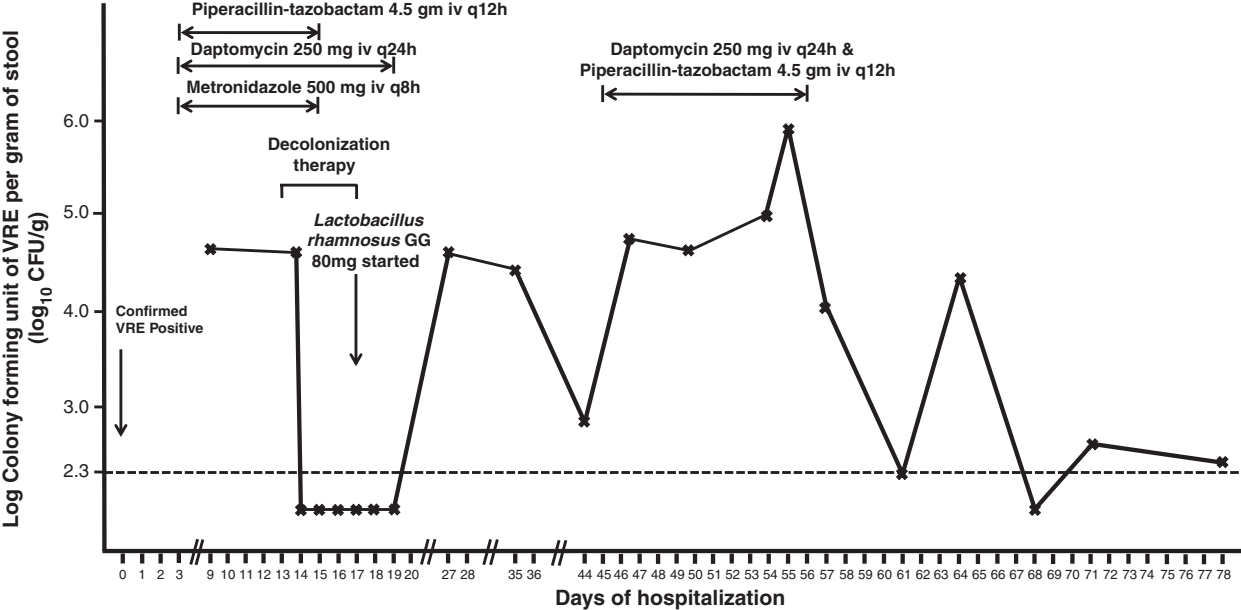

Supplement: Supplementary file 2 — Authors’ original file for figure 2 [file 12879_2014_3833_MOESM2_ESM.pdf]

Log Colony forming unit of VRE per gram of stool  
(log<sub>10</sub> CFU/g)

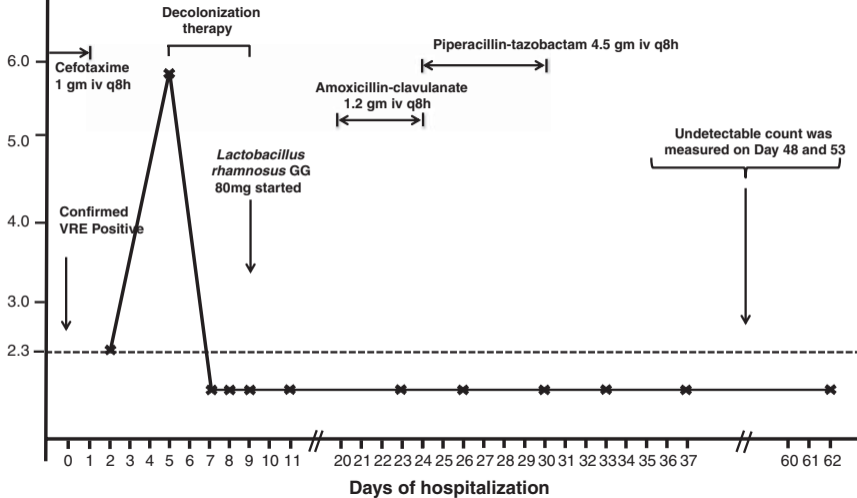

Supplement: Supplementary file 3 — Authors’ original file for figure 3 [file 12879_2014_3833_MOESM3_ESM.pdf]

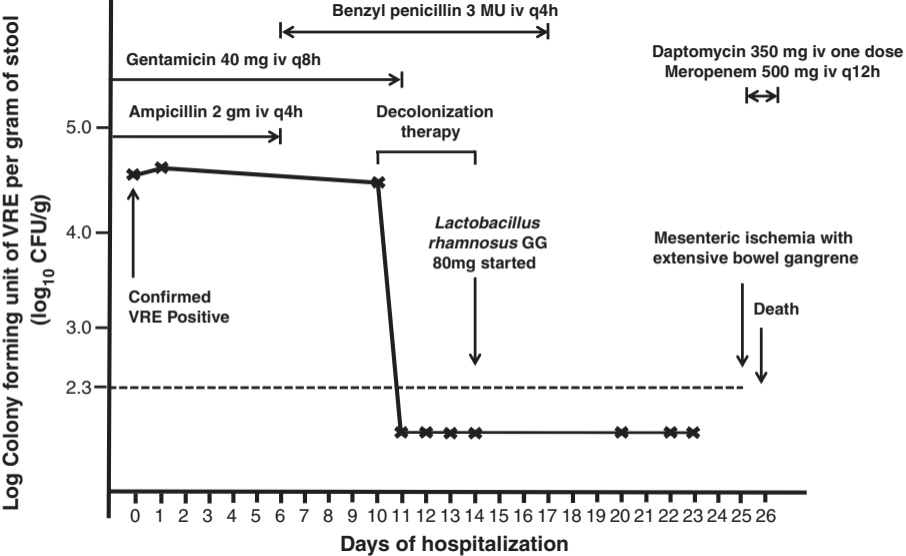

Supplement: Supplementary file 4 — Authors’ original file for figure 4 [file 12879_2014_3833_MOESM4_ESM.pdf]
